# The Effects of Dance Movement Therapy in the Treatment of Depression: A Multicenter, Randomized Controlled Trial in Finland

**DOI:** 10.3389/fpsyg.2020.01687

**Published:** 2020-08-12

**Authors:** Katriina Hyvönen, Päivi Pylvänäinen, Joona Muotka, Raimo Lappalainen

**Affiliations:** Department of Psychology, University of Jyväskylä, Jyväskylä, Finland

**Keywords:** dance movement therapy, randomized controlled trial, adults, depression, Beck Depression Inventory, Clinical Outcomes in Routine Evaluation—Outcome Measure, The Symptoms Check List-90

## Abstract

This multicenter research investigates the effects of dance movement therapy (DMT) on participants diagnosed with depression. In total, 109 persons participated in the study in various locations in Finland. The participants were 39 years old, on average (range = 18–64 years), and most were female (96%). All participants received treatment as usual (TAU). They were randomized into DMT + TAU (*n* = 52) or TAU only (*n* = 57). The participants in the DMT + TAU group were offered 20 DMT sessions twice a week for 10 weeks in addition to standard care. The measurement points included pretreatment measurement at the baseline, posttreatment measurement at the end of the intervention, and a follow-up measurement 3 months afterward. The observed effects of the intervention among participants in the DMT+TAU group were a greater reduction in depression and in indicators of physical and psychological distress in comparison to the participants who received TAU-only. At the 3-month follow-up, the corrected between-group effect sizes (ESs) were medium and in favor of the DMT + TAU group (*d* = 0.60–0.72). These results are in line with the increasing number of research studies showing the benefits of DMT intervention among participants with depression, and these results indicate that DMT may improve the effectiveness of standard care.

**Clinical Trial Registration:** www.ClinicalTrials.gov, identifier NCT04421651.

## Introduction

The debilitating condition of depression is the leading cause of ill health and disability in the adult population worldwide, and the prevalence of depression has increased between 2005 and 2015 by over 18% (WHO, 2017^1^). Depression is therefore a considerable burden to families and societies across the world. The same trend can be seen in Finland, where mental health problems have been the leading cause of work disability retirements since year 2000, and among the mental health problems, depression has been the most prevalent (Findikaattori, 2019^2^).

There is increasing evidence of the effectiveness of dance movement therapy (DMT) with a range of client groups and conditions (see reviews by Ritter and Low, 1996; Cruz and Sabers, 1998; Koch et al., 2014, 2019), including the reduction in the symptoms of depression (Jeong et al., 2005; Koch et al., 2007; Röhricht et al., 2013; Punkanen et al., 2014; Meekums et al., 2015; Pylvänäinen et al., 2015; Karkou et al., 2019). DMT is a form of creative arts therapy, which aims to integrate physical, emotional, cognitive, and social aspects into psychological treatment (Stanton-Jones, 1992; Meekums, 2002; EADMT Ethical Code, 2010^3^). DMT works in the sphere of embodiment and the integral relationship that the body has to the functioning of the mind (Homann, 2017; Payne et al., 2019). The methods involve shifting attentional states and moving in deeper relationship to self and others, for example, by fostering attuned interoceptive sensing, somatic awareness, and interactive dance (Homann, 2017). In DMT, also labeled as dance movement psychotherapy, the two central constructs are the healing power of conscious movement and the embodied creative experience (Payne, 1992; Caldwell, 2017). In the work during the therapy session, the careful balancing of breathing, moving/expressing, and feeling/sensing is essential (Caldwell, 2017).

Dance movement therapy engages the patients in physical and verbal exploration of their experiences generated in movement-based interaction. In the DMT research, there have been only three previous randomized controlled trials involving participants with depression, and the number of participants in these studies has ranged from 31 (Röhricht et al., 2013) to 72 (Xiong et al., 2009) participants (for reviews, see Meekums et al., 2015; Karkou et al., 2019). In the present research, we aimed to respond to this research gap by including a larger number of participants (*N* = 109) than had been done in previous studies utilizing randomized controlled trials in research designs. Our study has generated further information about the effectiveness of DMT groups in the rehabilitation of depression, as it was conducted in five cities across Finland. This type of multicenter research can provide a realistic picture of the typical practice of a range of dance movement therapists working with participants in various geographic locations.

### Depression

Depression is a psychiatric syndrome that is described by 10 symptoms: low mood, a loss of interest or pleasure, fatigue, decrease in self-confidence or feelings of worthlessness, excessive feelings of guilt and self-blame, thoughts about suicide or self-harm, self-harming behavior, decreased concentration, either slowed or agitated movement, sleep disturbances, and changes in appetite or weight (International Classification of Diseases, 10th Revision diagnostic system; Finnish Current Care Guidelines, 2016^4^). To fulfill the criteria for the diagnosis, a person needs to have at least two of the first three symptoms and in total four or more of these symptoms. These symptoms need to be present over a 2-week period with the symptoms being considered to cause clinically significant impairments in functioning. In Finland, 6.5% of the population have depressive states during a year (Pirkola et al., 2005).

The acute treatment of mild and moderate depression involves antidepressants or psychotherapies, and their combination is recommended in the Finnish Current Care Guidelines^4^. The treatment of mild and moderate depressive states is focused on antidepressants or psychotherapy, and the combination of medication and psychotherapy has been shown to be most effective. In severe and psychotic depression, the combination of treatment modalities is emphasized. The aim of psychotherapy is typically to promote recovery and functioning. Psychotherapies can include various evidence-based, short- or long-term applications of cognitive, behavioral, psychodynamic, interpersonal, acceptance and commitment, and resource- and solution-focused therapies. Some of the therapy approaches may include applications of methods from creative arts therapies. Treatment programs in hospital units, day hospitals, and outpatient psychiatric clinics may provide some physical activity options and, sometimes, also creative arts therapies, such as art therapy, music therapy, and DMT. Furthermore, DMT can be an alternative for clients who want to process experiences through not only verbal articulation but also bodily experiences.

### Description of the Dance Movement Therapy

In this research, we refer to DMT, but DMT is also called dance therapy, dance movement psychotherapy, movement psychotherapy, dance/movement therapy, or dance-movement therapy (Meekums et al., 2015). The European Association Dance Movement Therapy^5^ has defined DMT as “the therapeutic use of movement to further the emotional, cognitive, physical, spiritual, and social integration of the individual.” Dancing is considered central to DMT and is seen as body movement, creative expression, and communication. A dance movement therapist analyzes body movement to assess clients and devise movement interventions based on the basic premise of DMT: the interrelations of the mind and body as well as emotional states and relationships.

Dance movement therapy can be practiced by individuals (one-to-one) or in groups with a range of client populations from children to the elderly. The aim of DMT practice is to integrate physical, emotional, cognitive, and social aspects in the treatment (e.g., Stanton-Jones, 1992; Meekums, 2002). DMT focuses on embodied experiences emerging through body movements, expressions, and interactions with the environment (Koch and Fischman, 2011). In addition, participants can benefit from the physical responses related to exercise (Meekums et al., 2015; Karkou et al., 2019). In DMT, the participants can tune their awareness to these embodied experiences of their own self in a group therapy setting with others. This can support and strengthen therapeutic alliances, when experiences are reflected and communicated in words (Pylvänäinen, 2018). Zubala (2013) suggests that central to the treatment of depression in arts therapies is the cultivation of relatedness to oneself and to others. Also, fostering attentiveness and connection to body sensations develops one’s body image, self-relation, and action modulation. This can result in an improved perception of one’s body image, that is, increased awareness of the embodied phenomena, a more accepting and more neutrally descriptive relation to one’s body, and improved tolerance of others (Pylvänäinen and Lappalainen, 2018). Therapeutically, the creative change process is the core component of DMT, involving movement metaphors that surface in clients’ postures or movement experiences (Ellis, 2001; Meekums, 2002; Meekums et al., 2015; Karkou et al., 2019). Metaphors can yield information about a client and shape new perspectives, aiding in working through problematic experiences (Ellis, 2001).

Meta-analyses have shown that DMT and the therapeutic use of dance can be effective with a range of disorders, including psychiatric symptoms, autism, eating disorders, and stress (Ritter and Low, 1996; Cruz and Sabers, 1998; Koch et al., 2014, 2019). Furthermore, DMT has been shown to improve well-being, mood, affect, quality of life, body image, and interpersonal competence, as well as reducing clinical symptoms, such as depression and anxiety (Koch et al., 2014). The research thus far has systematically shown, albeit with smaller numbers of participants, that DMT can be an effective intervention in the treatment and rehabilitation of depression (Jeong et al., 2005; Koch et al., 2007; Röhricht et al., 2013; Punkanen et al., 2014; Pylvänäinen et al., 2015; Karkou et al., 2019). In addition, in the Finnish context, previous studies (Punkanen et al., 2014; Pylvänäinen et al., 2015; Pylvänäinen and Lappalainen, 2018) have shown that DMT is effective in alleviating depressive symptoms and improving mood. Participants in DMT groups have reported, for instance, an increase in their secure attachment style measured by the Relationship Questionnaire (Punkanen et al., 2014) and described that after DMT group therapy, they tolerated others better, were more active in social interaction, and felt less tense and more trusting of their own body (Pylvänäinen and Lappalainen, 2018). Specifically, DMT groups can have positive influences on the body image of participants with depression (Pylvänäinen and Lappalainen, 2018). Participants’ body image changed toward a more positive relatedness to their own body. The positive change in body image from the pre- to posttreatment measurements of the groups predicted a more significant reduction in depression and other symptoms from the pretreatment measurement to the 3-month follow-up. Specifically, a more positive change in body image predicted better mood.

A Cochrane review of the effects of DMT on depression compared the effects of DMT with no treatment and with standard care, psychological interventions, drug treatment, and other physical interventions (Meekums et al., 2015). Only three studies met the Cochrane review inclusion criteria, covering 147 participants in total. DMT was found to have reduced symptoms of depression at the follow-up measure more so than standard care among adults with depression in two of the studies. However, due to the low methodological quality of the studies and small sample size, no firm conclusions regarding the effectiveness of DMT can be drawn (Meekums et al., 2015). A recent meta-analysis of DMT found that studies with moderate to high methodological quality demonstrate strong support for the effectiveness of DMT concerning depression (Karkou et al., 2019). This meta-analysis took into account studies that had controlled designs and involved pre- and posttreatment testing. The preliminary results of the present study were included in that meta-analysis by Karkou et al. (2019). This research now presents the detailed findings that have been referred to in the published meta-analysis. The research responds to the need for larger randomized controlled trials regarding the effectiveness of DMT in the treatment of depression that could provide more reliable information for use in developing the treatment and rehabilitation of depression.

### Present Study

As depression has become a globally recognized problem in society, it is crucial to investigate alternative treatment methods and choices available for clients to engage in as therapy. In this research, we utilized a multicenter study design to investigate the effects of group DMT on depression symptoms among participants who have been diagnosed with clinical depression. In our research, we compare individuals who took part in a DMT intervention with those who received treatment as usual (TAU). Participants in the DMT group also continued their TAU in their psychiatric outpatient clinic, occupational health service, or other health service provider responsible for the standard care. Our research questions were as follows:

1. Is there a change in depression symptoms [Beck Depression Inventory (BDI)] and other psychological and physical symptoms [Clinical Outcomes in Routine Evaluation—Outcome Measure (CORE-OM) and The Symptoms Check List-90 (SCL-90)] among participants in the DMT treatment group at the three measurement points: before the intervention period (pretreatment measurement), after the intervention (posttreatment measurement), and after the 3-month follow-up period (follow-up measurement)?
2. Is the change in symptoms different among participants in the DMT treatment group with standard care (DMT + TAU) compared with those participants who receive standard care only (i.e., the control group; TAU only)?

Previous research has shown that DMT alleviates symptoms of depression and other psychological and physical problems (e.g., Jeong et al., 2005; Koch et al., 2014, 2019; Punkanen et al., 2014; Pylvänäinen et al., 2015; Karkou et al., 2019). Based on those findings, we expected that the reduction in depression and other psychological and physical symptoms among participants in the DMT treatment group would be significantly greater than the change in the control group.

## Methods

### Recruitment Procedure

**FIGURE 1:**
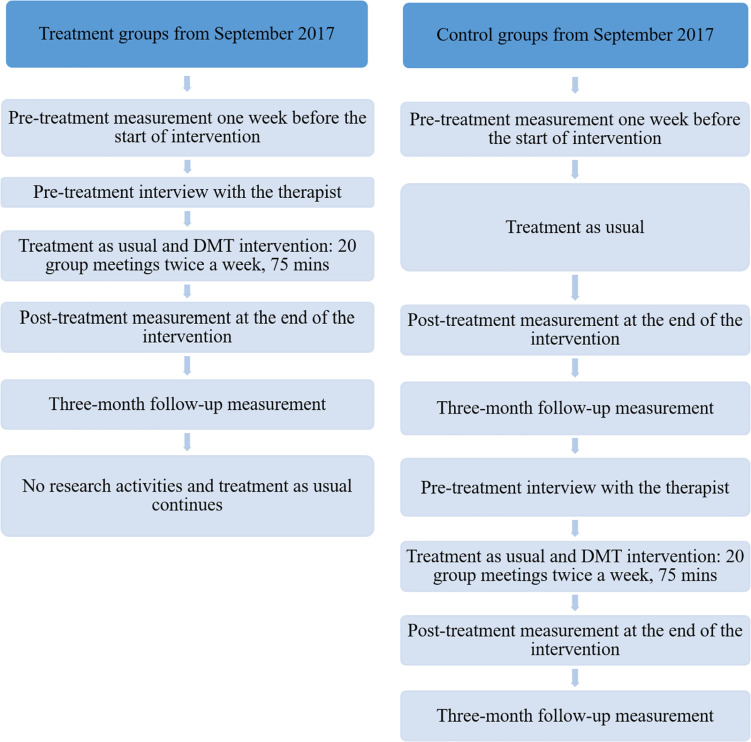
The progress of participants through the trials.

The present research is part of a larger intervention study funded by the Finnish Social Insurance Institution on the effectiveness of DMT in treating depression. The DMT group sessions took place in seven cities across Finland. This research focuses on the data collected from the randomized controlled trials that were performed in larger cities in the Greater Helsinki (three groups), Tampere (one group), Jyväskylä (two groups), and Joensuu (two groups). In the smaller cities, the randomization of the participants was not possible due to the small number of participants there; hence, those non-randomized participants were excluded from this research. The participants in this research were randomly allocated to parallel groups: either in the treatment group (DMT + TAU) or the control group (TAU only). Participants in the treatment group participated in the DMT sessions in the autumn of 2017. In addition, the participants in the control group had the opportunity to attend DMT sessions in the spring of 2018. Figure 1 shows the progress of participants through the trials.

Participants were recruited through public and private mental health services, which included outpatient clinics, student health services, occupational health services, and psychiatrists in private health clinics. The possibility to participate in the research and DMT groups was also advertised in local newspapers and through social media. Persons interested in taking part in the study contacted the researchers by phone or email to book a time for a screening interview conducted over the phone during the spring and summer of 2017. The researchers interviewed altogether 235 persons over the phone.

The aim of each screening interview was to check if the inclusion criteria of the study were met by the participant. First, the participant needed to have a diagnoses of depression by a psychiatrist or general practitioner (International Classification of Diseases—diagnostic system). Second, the researchers checked that the participants were working age (18–65 years of age) and fulfilled the criteria for rehabilitative psychotherapy in Finland (KELA, 2020^6^): at least 3 months of relevant treatment since depression was diagnosed; the participants’ ability to work or study is impaired by depression; and a psychiatrist has determined that rehabilitative psychotherapy is necessary to improve or support the participant’s ability to work or study. The exclusion criteria were (1) active suicidal ideation, (2) psychotic symptoms, and (3) substance misuse that is on a critical level (Audit questionnaire scores above 10). Also, interviewees who had pain-related problems that restricted daily life or were pregnant were not admitted to the study. Study participants were given an information leaflet regarding the participation in the study and were asked to sign a consent form. The board of research ethics at the Central Finland Health Care District has affirmed the present research with a favorable statement (Dnro 8U/2016). The research is registered and posted on the ClinicalTrials.gov public website (identifier: NCT04421651).

In total, 118 participants were randomized into the treatment and control groups. The coordinating researchers responsible of the data collection used the SPSS program to randomly select 59 participants in the treatment and 59 participants in the control groups. The contact details of the participants in the treatment group were forwarded to the therapists working in different locations. The therapists facilitating the treatment were responsible in organizing suitable locations for their group. The therapists were in contact with the participants to inform them about the dates, times, and location of the DMT groups and organize pretreatment interview.

The researchers contacted the participants in the control group to inform that they had been randomly selected to the control group. Nine of these participants dropped out before the pretreatment measurement of the research and start of the DMT group sessions for reasons such as changes in life circumstances since the screening interview (e.g., moved town) or the times of the group sessions not having been suitable. This study focuses on those participants (*n* = 109) who were randomly allocated to either the treatment group (*n* = 52) or control group (*n* = 57). At the pretreatment measurement, before the DMT intervention commenced, all participants responded to an electronic survey regarding their psychological and physical symptoms related to depression. The posttreatment measurement was conducted immediately after the intervention and the response rate was 84% (92 participants), comprising 81% of participants from the treatment group and 88% from the control group. The follow-up measurement was conducted 3 months after the intervention, and the response rate was 71% (77 participants), consisting of 73% of participants from the treatment group and 68% from the control group. All participants continued their usual treatment during the research. The TAU included antidepressant medication among 56% of participants. Almost 68% of participant also had individual sessions with a health care professional once in 4 weeks or more frequently. The demographical information of the participants and other treatment they received during the research are shown in Table 1.

**TABLE 1:** Demographic information on participants in the treatment and control groups.

	**Treatment group**	**Control group**	**Total**
	
	*n* = 52	*n* = 57	*N* = 109
**Sex (%)**			
Female/male	98.1/1.9	94.7/5.3	96.3/3.7
Age in years			
Average	41.8	36.5	39.03
Min.	18	18	18
Max.	63	64	64
**Education (%)**			
Comprehensive school	7.7	5.3	6.4
Secondary education	30.8	36.8	33.9
Vocational school	11.5	10.5	11.0
Lower university degree	26.9	22.8	24.8
Higher university degree	21.2	17.5	19.3
Other (e.g., Ph.D.)	1.9	7.0	4.6
**Employment situation (%)**			
Full-time employment	21.1	14.0	17.4
Part-time employment	17.3	5.3	11.0
Unemployed	5.8	14.0	10.1
Disability allowance	5.8	8.8	7.3
Studying	19.2	29.8	24.8
Other (e.g., sick leave, freelance, scholarship)	30.8	28.1	29.4
**Antidepressant medication (%)**			
Yes/no	65.4/34.6	47.4/52.6	56.0/44.0
**Other treatment (%)**			
Individual sessions every 1–2 weeks	28.6	33.3	31.1
Individual sessions every 3–4 weeks	34.3	38.5	36.5
Individual sessions every 5 weeks or less frequently	22.9	15.4	18.9
Weekly treatment group	2.9	12.8	8.1
Self-initiated hobbies and leisure groups	25.7	35.9	31.1
**Previous depression episodes (%)**			
Yes/no	73/27	70/30	71.6/28.4

The nature of the missing information (i.e., non-responses) in the data was analyzed using Little’s missing completely at random test. The missing completely at random test was valid and showed that information was missing at random: *x*^2^(21) = 12.884, *p* = 0.913. More specifically, 69% (*n* = 75) of participants responded at three measurements, whereas 31% (*n* = 34) responded at one or two measurements. Differences between these groups in age, use of antidepressant medication, and previous depression episodes as well as outcome measures (BDI, CORE-OM, and SCL-90) at pretreatment measurement were analyzed with independent *t*-tests and chi-square tests. No differences were found between respondents and non-respondents in relation to the age, use of antidepressant medication, previous depression episodes, or outcome measures.

### Dance Movement Therapy Treatment

The participants who were randomly assigned to the treatment group received a DMT group intervention that took place twice a week for 10 weeks starting in September 2018. In total, the intervention period included 20 sessions, which were 75 min each. The duration and frequency of the treatment period was similar to the one used in the study by Punkanen et al. (2014), but sessions were 15 min longer than in their study. The number of participants in each group varied between 4 and 8. There were altogether eight DMT groups in different cities across Finland, and these were facilitated by eight trained dance movement therapists whose first degrees varied (e.g., health care professionals and teaching degrees). In addition, all therapists attended the 12-day intervention training before the beginning of the group sessions and were provided supervision during the intervention period. The potential advantages, disadvantages, and unintended effects were outlined in the information leaflet given to the participants. The participants were informed that it is possible that they do not benefit from the treatment. The participants were also told that the DMT practices are safe, participation does not require physically demanding exercises, and the groups are facilitated by experienced DMT practitioners. The disadvantages of participation included for the participants the time required for responding to the surveys and that they were attending the group on their own time. The unintended effects of participation could relate to the process of working through difficult feelings or experiences in therapy. The participants were encouraged to discuss with the therapist, researchers, or their doctor regarding their experiences in the group if they were uncertain about continuing in the group.

The DMT treatment was based on an integrative perspective on the treatment of depression, and different theories and concepts were applied from psychotherapy approaches: psychodynamic, cognitive, solution-focused, trauma therapy, object relations, interpersonal theories, and relational–cultural theories. This type of integrative approach is typical of DMT. The DMT methods used included dance and movement improvisations, mindfulness practices, use of props (e.g., fabrics and balls), and reflection through drawing, writing, and discussion. The group meetings consisted of orientation within the group, thematic working, and closure. The themes that these groups were built upon were based on previous DMT research regarding the treatment of depression: goals, movement options and boundaries, body awareness and resources, symbols, safety, regulation, expressing emotions, body narrative, playfulness, needs, being and doing, agency, and processing the time span of the group and one’s own life (for more details, see Punkanen et al., 2014; Pylvänäinen et al., 2015).

### Outcome Measures

Beck Depression Inventory I (Beck et al., 1961, 1988, 1996) measures depressive symptoms with 21 items and is frequently used in clinical assessment. Each item is scored 0–3, and sum scores are calculated on the basis of participants’ responses. The total sum score can range from 0 to 63. A score from 0 to 9 indicates no or very few depressive symptoms, from 10 to 18 indicates mild depression, from 19 to 29 moderate depression, and from 30 to 63 severe depression. The Cronbach alphas for the BDI-I were 0.86 for pretreatment, 0.91 for posttreatment, and 0.91 for follow-up measurements.

Clinical Outcomes in Routine Evaluation—Outcome Measure (Evans et al., 2002; Barkham et al., 2005; Connell et al., 2007) measures participants’ mood and distress. The CORE-OM is sensitive to change in symptoms and can therefore be used to assess the clinical effectiveness of therapy (Evans et al., 2002). The CORE-OM comprises 43 items and four dimensions of well-being, problems, life functioning, and risk for aggressive/suicidal behavior. Each item is scored from 0 to 4. CORE-OM items are summed and then divided by the number of answered items, which is then multiplied by 10, that is, the total scores ranges from 0 to 40. The higher the score, the more severe symptoms the respondent has. Between the general and clinical populations, the clinical cutoff point is 10 points (Connell et al., 2007). The CORE-OM all-items score has a correlation of 0.85 with the BDI-I and 0.88 with the SCL-90-revised version (Evans et al., 2002). The Cronbach alphas for the CORE-OM were 0.77 for pretreatment, 0.83 for posttreatment, and 0.79 for follow-up measurements.

The Symptoms Check List-90 (Holi, 2003) uses 90 items to measure nine primary symptomatic dimensions, including somatization, obsessive–compulsiveness, interpersonal sensitivity, depression, anxiety, hostility, phobic anxiety, paranoid ideation, and psychoticism. The level of distress is scored on a scale from 0 to 4. An average score of the 90 items is calculated, which represents the severity of the participant’s symptoms, that is, the Global Severity Index (GSI). The higher the GSI, the more symptoms the participant has. In the community sample, the GSI mean was 0.60 and in the patient sample, 1.56 (Holi, 2003). The Cronbach alphas for the SCL-90 were 0.96 for pretreatment, 0.98 for posttreatment, and 0.98 for follow-up measurements.

The following demographic characteristics were taken into consideration in the analyses: age in years (continuous), use of antidepressant medication (1 = no, 2 = yes), and previous depression episodes (1 = no, 2 = yes).

### Statistical Analysis

First, between-group differences in demographic data were analyzed with independent *t*-tests and chi-square tests. Second, the effects of the intervention were analyzed using hierarchical linear modeling (HLM) in Mplus (version 7) (Muthén and Muthén, 2012). By means of HLM with the full information maximum likelihood estimation method, we can use all of the available information and include all the participants who started the study in our analyses. The missing data in the HLM and full information maximum likelihood are assumed to be missing at random. The between-group difference in the change of symptoms was analyzed with the Wald test. If the interaction between group × time was statistically significant, the group differences were tested for the intervention period (pretreatment to posttreatment measurement) and follow-up period (posttreatment to follow-up measurement) separately. The participants’ use of antidepressant medication was controlled for in the analyses. Also, the within-group differences in symptoms were analyzed with the Wald test.

Third, the effect sizes (ESs) were calculated. The between-group ES were calculated at the pretreatment, post-treatment, and follow-up measurement times by dividing the difference between the treatment group mean and the control group mean by the pooled standard deviation (SD) of the conditions. Due to possible differences between groups at the pretreatment measurement point, between-group ES differences at the posttreatment and follow-up measurements were corrected by taking into account their baseline differences. Thus, corrected between-group ES are reported here. The within-group ES was calculated for both pre- and posttreatment measurements by dividing the mean change from the pretreatment measurement by the combined (pooled) SD (Feske and Chambless, 1995; Morris and DeShon, 2002). A *between-group* ES of 0.2 was considered small, 0.5 medium, and 0.8 large. And *within-group* ES of 0.5 was considered small, 0.8 medium, and 1.1 large (Roth and Fonagy, 1996).

The effectiveness of the DMT intervention was also examined with the Reliable Change Index (RCI; Jacobson and Truax, 1991) of the BDI-I scores. The RCI can help determine whether the participants’ change is statistically reliable and not due to a measurement error. The RCI indicates the clinical significance of the change in depression symptoms and whether the participants can be considered to have improved during the intervention. The RCI classification accounts for whether a participant’s extent of improvement has passed the weighted midpoint between the means of the general population and the clinically symptomatic one (Jacobson and Truax, 1991; Seggar et al., 2002); if so, the participant would be classified as recovered. For the BDI-I, the weighted midpoints were 13.13 for the treatment group and 13.89 for the control group. Participants whose BDI-I scores were above the weighted midpoint were included in the RCI calculations. Participants whose BDI score was below the cutoff point at the pretreatment measurement time were excluded from the classification. If a participant’s RCI is below –1.96 and passes through the cutoff point, the participant is classified as *recovered*. If the RCI is below –1.96 but does not pass the cutoff point, the participant is classified as *improved*. If the participant’s RCI is between –1.96 and 1.96, the participant is classified as *unchanged*. If the participant’s RCI is above 1.96, the participant is classified as *deteriorated*. We examined, using analysis of variance and chi-square tests, whether there were differences in demographic characteristics and BDI-I scores at the pretreatment measurement time between participants with different RCI classifications.

We calculated *post hoc* power in BDI with the MPlus using Monte Carlo Simulations in which the number of replications was 10,000. The *post hoc* power of the Wald test (time × group) was 0.991. The power was 0.840 for the parameter, in which the change between pre- and posttreatment measurements was explained by the group. The power was 0.499 for the parameter, in which the change between posttreatment and follow-up measurements was explained by the group.

## Results

### Descriptive Results

The participants in the treatment and control groups were compared in terms of demographic characteristics to test whether there were differences between groups at the pretreatment measurement point. The participants in the treatment group were older (*M* = 41.8 years) than the participants in the control group (*M* = 36.5 years), *t*(107) = 2.25, *p* > 0.05. There were no differences between the groups in the representation of sex, level of education, employment situation, use of antidepressant medication, other treatments, or previous depressive episodes.

Table 2 shows the correlation of age, use of antidepressant medication, and previous depression episodes in relation to the outcome measures used. Of the demographic characteristics considered, only the use of antidepressant medication was related to lower scores on the BDI-I and CORE-OM measures and also to higher age. Therefore, we chose to control for the use of medication in the subsequent analyses.

**TABLE 2:** Correlation coefficients for the demographic variables and the outcome variables at the pretreatment measurement and their change between the pretreatment and follow-up measurements.

Variables	1	2	3	4	5	6	7	8	9
**Depression (BDI-I)**									
1. Pre-treatment measurement	–								
2. Pre to Follow-up	0.158	–							
**Psychological distress (CORE-OM)**									
3. Pre-treatment measurement	0.809***	0.083	–						
4. Pre to Follow-up	0.078	0.790***	0.209	–					
**Psychiatric symptoms (SCL-90)**									
5. Pre-treatment measurement	0.712***	0.014	0.827***	0.047	–				
6. Pre to Follow-up	–0.028	0.729***	0.016	0.774***	0.102	–			
**Demographic characteristics**									
7. Age in years	0.046	0.206	–0.007	0.089	–0.032	0.06	–		
8. Medication (1=no; 2=yes)	−0.273**	0.060	−0.365***	–0.156	–0.188	0.119	0.189*	–	
9. Previous episodes (1=no; 2=yes)	–0.036	–0.140	–0.042	–0.204	0.072	–0.113	0.047	–0.109	–

*p < 0.05; **p < 0.01; ***p < 0.001.

### Levels and Changes in Symptoms Across the Measurements

**FIGURE 2:**
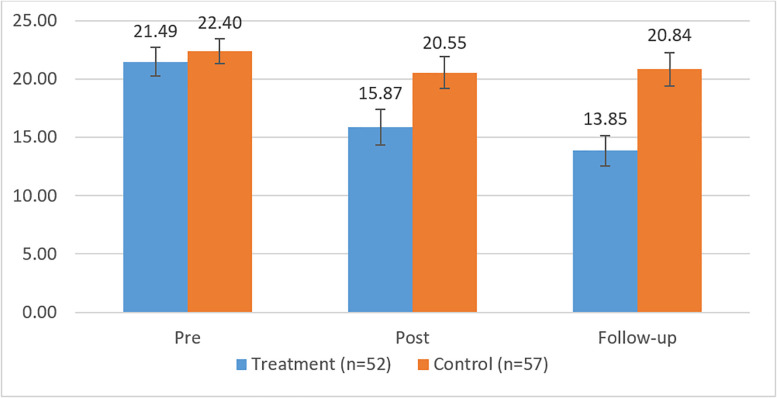
Means and error bars BDI-I in the treatment and control groups between pre- and follow-up measurement.

**FIGURE 3:**
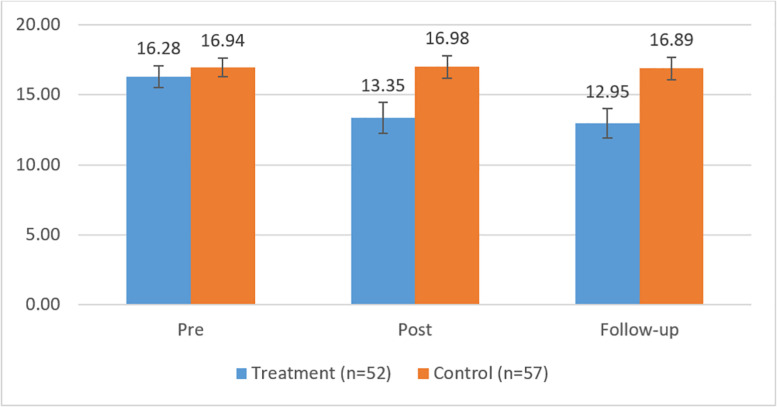
Means and error bars in CORE-OM in the treatment and control groups between pre- and follow-up measurements.

**FIGURE 4:**
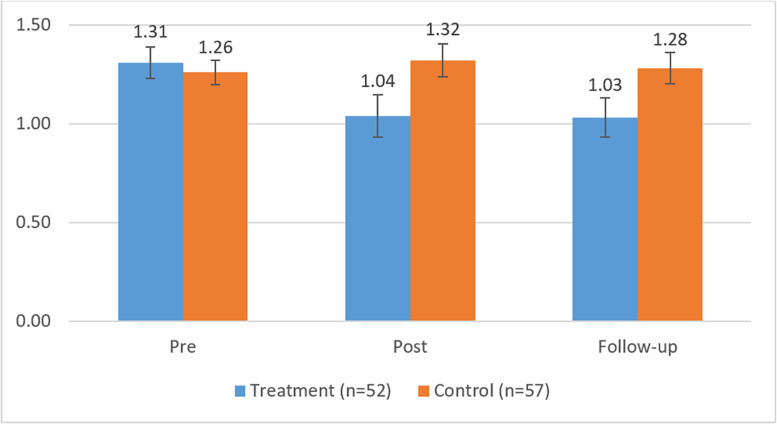
Means and error bars in SCL-90 in the treatment and control groups between pre- and follow-up measurements.

The means and SD of the outcome measures, as well as the corrected between-group ES and the results from the Wald tests, are shown in Table 3 for the three measurement points. First, it can be noted from Table 3 that, at the pretreatment measurement time, there were no statistically significant differences between groups in the BDI-I, CORE-OM, or SCL-90, indicated by the estimates of the Wald test. Furthermore, in line with our expectations, we observed significant differences between participants in the treatment and control groups with respect to the outcome measures for the period from the pretreatment to follow-up measurement. However, no differences in the change of outcome measures were observed for the posttreatment to follow-up measurement time span between the participants in the treatment and control groups, that is, the levels of symptoms remained stable during the follow-up period. Figures 2–4 show the means and error bars of outcome measures for the treatment and control groups at the three measurement points.

**TABLE 3:** Means and standard deviations of outcome measures in treatment and control groups: results of the Wald tests, including estimates and corrected between-group effect sizes.

Measurement	Pretreatment	Posttreatment	Follow-up	Wald test time	Wald test time × group
					
*BDI*					11.69**
1. Treatment group	21.49 (8.86)	15.87 (9.71)	13.85 (8.33)	35.65***	
2. Control group	22.40 (8.00)	20.55 (9.98)	20.84 (10.27)	5.26, ns	
Estimates for group comparisons	–0.12, ns	–3.94*^1^	–2.16^2^, ns		
Between-group ES	0.11	0.45^1^	0.72^3^		
*CORE-OM*					9.87**
1. Treatment group	16.28 (5.75)	13.35 (7.26)	12.95 (6.77)	15.36***	
2. Control group	16.94 (5.13)	16.98 (5.87)	16.89 (5.56)	0.01, ns	
Estimates for group comparisons	0.06, ns	–3.34**^1^	–0.52^2^, ns		
Between-group ES	0.12	0.55^1^	0.60^3^		
*SCL-90*					9.21*
1. Treatment group	1.31 (0.58)	1.04 (0.73)	1.03 (0.67)	18.55***	
2. Control group	1.26 (0.46)	1.32 (0.61)	1.28 (0.55)	1.11, ns	
Estimates for group comparisons	0.09, ns	–0.36**^1^	0.045^2^, ns		
Between-group ES	0.10	0.64^1^	0.60^3^		

^1^Pre–Post; ^2^Post–Follow-up; ^3^Pre–Follow-up; *p < 0.05; **p < 0.01; ***p < 0.001.

The measurement time had a main effect on the BDI, CORE-OM, and SCL-90, and the symptoms they measured showed a significant decrease across the measurement times among participants in the DMT group. Interaction effects were observed between time and group, indicating that the symptoms measured by the BDI, CORE-OM, and SCL-90 decreased more among the participants in the treatment group compared with those in the control group, wherein no decrease in symptoms was observed.

To assess the size of the intervention effects, the corrected ES were analyzed. At the follow-up measurement point, between-group ES showed medium differences for all the measures (*d* = 0.60–0.72). In the treatment group, the within-group ES were small between the pretreatment and posttreatment measurements with respect to the BDI-I (*d* = 0.60) and close to small for the CORE-OM (*d* = 0.44) and SCL-90 (*d* = 0.42). Between the pretreatment and follow-up measurements, the within ES were medium (*d* = 0.89) with respect to the BDI-I and small or close to small with respect to the CORE-OM (*d* = 0.53) and SCL-90 (*d* = 0.45).

In the control group, the within-group ES were very small (BDI-I, *d* = 0.17; CORE-OM, *d* = –0.01; SCL-90, *d* = –0.11) between the pretreatment and posttreatment measurements. The within-group ES were also small between the pretreatment and follow-up measurements (BDI, *d* = 0.21; CORE-OM, *d* = 0.01; SCL-90, *d* = –0.05). Thus, in the treatment group, the within-group ES varied between the pretreatment and follow-up measurements from 0.45 to 0.89 compared with that in the control group’s, -0.11 to 0.21.

The RCI classification was first analyzed for the participants in the DMT treatment group, whose BDI-I scores were above the cutoff point at the pretreatment measurement time and who had responded at the measurement points (*n* = 30). Of these participants, 13 (43.3%) had either recovered (*n* = 9; 30%) or improved (*n* = 4; 13.3%). In addition, 17 (56.7%) participants were classified as unchanged, and none had deteriorated. There were no differences between the participants with different RCI classifications regarding age, use of antidepressant medication, or previous depression episodes. However, the participants who were classified as improved had reported significantly higher levels of symptoms in the BDI-I measure at the study baseline (*M* = 30.8) compared with the participants who recovered (*M* = 22.56) or remained unchanged (*M* = 21.9), *F*(2,27) = 3.587, *p < 0*.05.

In the control group (*n* = 35), five (14.3%) participants had either recovered (*n* = 2; 5.7%) or improved (*n* = 3; 8.6%). A large majority of the participants in the control group remained unchanged (*n* = 29; 82.9%), and one participant deteriorated (*n* = 1; 2.9%).

## Discussion

The main finding of this study shows that for participants with depression, attending a DMT group, in addition to TAU, can reduce their depression symptoms, as well as other psychological and physical symptoms, more so than by receiving TAU only. In other words, adding a DMT group intervention to the usual treatment of depression improved the effectiveness of the depression treatment as observed across three measurement points that spanned from the beginning to the end of the DMT intervention plus a follow-up 3 months afterward. The participants with depression who are motivated to address personal experiences through movement and dance benefit from having the possibility to join DMT groups, and their symptoms decrease more than if they were just receiving their usual psychological and medical treatment. These findings are in line with previous research among smaller number of participants with depression in Finland (Punkanen et al., 2014; Pylvänäinen et al., 2015) as well as in international meta-analyses (Koch et al., 2007; Karkou et al., 2019). The research gives support that DMT groups can be a beneficial and cost-effective form of short-term group therapy. The participants who committed to the group were also likely to complete the group process, which was seen across a number of different therapists that were facilitating groups in various locations across Finland.

### Clinical Significance of the Changes in the Levels of Depression

The clinical significance of these differences in the levels of depression symptoms between the DMT + TAU and TAU-only groups at the different measurement times was considered through ESs and reliable clinical index classifications. First, it was noted that the corrected between-group ESs between the DMT + TAU group and TAU-only group, the latter having received standard care only, were medium at the end of the three-month follow-up period (*d* = 0.72) in favor of the DMT group. The between-group ES of over 0.70 suggests that about 66% of the DMT participants benefitted from the DMT treatment compared to 50% of the non-participants (see Cohen, 1988; Norcross and Lambert, 2010). The *number needed to treat* (NNT) indicates the number of participants who need to receive an intervention in order to achieve one success. An ES of 0.70 refers to an NNT of 3; that is, three participants needed to receive DMT to achieve one success compared to non-participants in a control group. In addition, the within-group ESs were considerably larger among participants attending to the DMT group. These ESs are comparable to the ESs found in other studies on the effectiveness of DMT in the treatment of depression in Finland that have involved treatment and control groups (Pylvänäinen et al., 2015). For instance, in the study by Pylvänäinen et al. (2015), among participants in Finland with diagnosed depression, the between-group ESs ranged from medium to large. The decrease in the mean level of symptoms examined with the BDI-I was similar (7.64 points) to what has been observed in the study by Punkanen et al. (2014) using a comparable DMT group intervention with 20 sessions. In their study, the reduction of the BDI-tested symptoms from the baseline to the post-treatment measurement was 11.17 points. Also, a shorter DMT intervention in Finland (Pylvänäinen et al., 2015) including 12 sessions indicated a favorable mean reduction in depression symptoms (measured with the BDI-II), that is, 10.11 points. The results of these three studies in Finland show that beneficial changes are seen in DMT interventions that are (1) 12 × 90 min sessions once a week (Pylvänäinen et al., 2015); (2) 20 × 60 min sessions twice a week (Punkanen et al., 2014); and (3) 20 × 75 min sessions twice a week in this research. When there are more group sessions and longer meeting time, the group may benefit from slowing down and working through group experiences at an unhurried pace.

The ESs observed in a meta-analysis of group psychotherapies showed larger ESs, averaging 1.03 at the post-treatment measurement time across studies (McDermut et al., 2006). Then, in a later meta-analysis (Cuijpers et al., 2010), group therapies were found to have an ES of 0.31. Clearly, there is variance. The difference in the ESs in our research compared to those in the study by McDermut et al. (2006) may also be explained by the different characteristics of the control group, which in their case included untreated participants. In our research, all of the participants in the control group received standard care during the research. The standard care that the participants in the treatment and control groups received included weekly or fortnightly individual sessions for around a third of the participants, and slightly over half of the participants attended individual sessions every 3 weeks or less frequently. In addition, 13% of the participants in the control group reported attending other treatment groups, whereas only 3% of the participants in the DMT group reported attending other treatment groups. However, it should be noted that this difference in standard treatment between the two groups in our study was not statistically significant. In the present study, in both the DMT + TAU and TAU-only groups, participants used antidepressants, and this can also play a role in the change patterns.

Furthermore, among the participants in the DMT+TAU group, over 40% were classified as recovered or improved on the basis of changes in the BDI-I scores calculated with the RCI classification. In comparison, only 14% of the participants in the TAU-only group recovered or improved. Demographic characteristics did not seem to be related to the extent to which participants benefitted from the DMT group treatment. Interestingly, the participants who were characterized as improved had reported a higher level of depression symptoms than other participants at the study baseline. These findings suggest that participants who have a fairly severe level of symptoms can also gain benefits from a DMT intervention. However, it should be noted that the number of participants classified as improved in the DMT group, four persons, was small. These findings for the ESs and RCI classifications suggest that the favorable changes observed in depression when the DMT was added to the TAU have clinical significance. It is notable that there are participants who are particularly motivated to work through difficult personal issues in therapy groups that include the opportunity to engage in creative movement and reflection on embodied experiences. This can be useful especially in the treatment of depression, which is a condition involving many body-related changes and difficulties (e.g., exhaustion, fatigue, loss of pleasure).

### Changes in Other Psychological and Physiological Symptoms

The reduction in depression symptoms among the DMT + TAU group was in line with changes seen in other measures of symptoms. A significant improvement in the DMT + TAU group over the TAU-only group was also observed in regard to psychological distress measured with the CORE-OM and psychiatric symptoms measured with the SCL-90. In fact, in the SCL-90, about a third of the items on the scale measure experiences that relate to somatization or arousal of the autonomous nervous system. It is therefore notable that a DMT intervention also relates to changes in the way in which participants experience their bodily felt symptoms (Pylvänäinen et al., 2015).

Overall, the reduction in various symptoms is in line with previous studies with a wide range of participants having different disorders (e.g., Koch et al., 2014, 2019; Meekums et al., 2015; Karkou et al., 2019). These previous reviews and studies confirm the benefits of DMT in relation to psychiatric symptoms as well as positive indicators of well-being, such as quality of life (Bräuninger, 2012) and vitality (Koch et al., 2007). A growing body of research suggests that DMT is an effective treatment for participants with depression. The mechanisms of change have been suggested to relate to participating in dance, which can be an art form as well as exercise, emphasizing one’s experience, sensation, presence, and expression through movement (Pylvänäinen et al., 2015; Karkou et al., 2019). For instance, a Cochrane review concluded that exercise can be as effective as antidepressants or psychological therapies in reducing the symptoms of depression (Rimer et al., 2012). At the same time, DMT can provide a therapeutic alliance in which the embodied relationships, symbolic unconscious material, and integration through reflection, movement, and creativity are central and focused upon (Karkou et al., 2019). It is also vitally relevant that, in DMT, it is possible to create involvement through moving in ways that are on the level of the participants’ physical capacity, current energy level, and motivation.

### Study Limitations and Future Research

There are several study limitations, as well as strengths, that should be taken into account when making inferences on the bases of these findings. The main limitation of the study is same-source bias, as only self-report measures were used. In future research, it may be useful to include also other physiologically oriented or movement-based measurements. Clearly, there is a physical component to DMT, so physiologically oriented or movement-based measurements would be relevant when investigating the effectiveness of DMT. In addition, less than 4% of participants were male, and therefore, the role of gender in benefiting the treatment could not be taken into account. It would be advisable to have more equally gender-balanced data in future research. On the other hand, the majority of patients with depression are female; thus, information specifically on the female patients’ experience and recovery is relevant (Koponen et al., 2018; WHO, 2020^7^).

Although the participants were randomly divided into the DMT + TAU and TAU-only groups, the comparisons of these groups showed that the participants in the DMT + TAU group were slightly older than those in the TAU-only group. We can see from the percentages of participants’ current employment status that, in the TAU-only group, there seemed to be more students and fewer participants who were in either full- or part-time employment; albeit, these differences were not statistically significant. Furthermore, there were no differences between the groups in their level of depression or other psychological or psychiatric symptoms at the baseline of the study. Participants’ age also seemed unrelated to the level of symptoms at the baseline and to the change in symptoms across the measurement times.

A longer follow-up period in future research may be important to gain further information about longer term effects of DMT. It could be that some effects take longer to surface. On the other hand, it is also possible that, for a number of participants, a 20-session group treatment is not sufficient to address the root causes and patterns of their depression and produce permanent improvements in their symptoms. For those reasons, on the basis of our current findings, we cannot yet draw firm conclusions about the stability of the positive trend seen in the reduction of DMT participants’ symptoms. A further limitation is that 29% of the participants who initially participated in the survey did not respond at the 3-month follow-up measurement time. In the statistical analyses, we were able to include all the participants who took part in the research. We do not have information from all of the non-respondents about the reasons why they had not responded or decided to drop out from the research. Several participants withdrew from the study due to changes in their life circumstances, such as in their employment situation or having started psychotherapy rehabilitation. However, there were also participants who did not inform us about their reasons for withdrawing from the study. It is therefore possible that some non-respondents’ health may have deteriorated and they felt too burdened to participate.

The strength of our multicenter research is that there were more participants in this study than in previous randomized controlled trials of DMT with participants diagnosed with depression in various geographical locations. The groups were facilitated by eight different therapists, and the group composition varied between 4 and 10 participants. Thus, there was likely to be similar heterogeneity in the skills and experience of the therapists as well as in the motivation and the severity of symptoms among the participants, as in real life therapy settings. The group processes could be different depending on the number and specific needs of the group members. The therapists adjusted the structured therapy process introduced in the training when necessary to respond to the needs and processes of the groups. In this type of multicenter research, we cannot conclude to what extent the results reflect the structured group process and what changes can be attributed to the responsiveness of the therapists running the group (see Stiles, 2013).

A higher number of participants would be needed to assess the differences between participants who either recover or improve during the intervention and also among those whose symptoms do not change. Out of 52 participants in the DMT group, only 30 participants responded at each measurement point, and their BDI-I scores were above the weighted cutoff points. In future research, it would be relevant to investigate, with a larger sample, the type of profiles that can be identified among participants in the DMT group. With a person-oriented approach (e.g., Wang et al., 2013; Bergman and Lundh, 2015), future research should identify profiles of participants who are as homogenous as possible within one profile as well as heterogenous between profiles. This type of analysis would be able to address what demographic characteristics and intervention-related factors predict benefitting from a DMT intervention.

The main findings of our research show that individuals participating in a DMT group treatment can benefit from this type of intervention in addition to their other treatment. In fact, the participants who were in the TAU-only group experienced, on average, no improvement in their symptoms. The DMT group sessions in the different cities were facilitated by a number of trained dance movement therapists, so these results are more likely to reflect DMT methods than the characteristics or skills of a specific therapist (Karkou et al., 2019). Our research thus encourages the application of creative and experiential group treatments, such as DMT, with the prospect of enhancing and thereby improving the effects of the depression treatment.

## Data Availability Statement

The datasets generated for this study are available on request to the corresponding author.

## Ethics Statement

The studies involving human participants were reviewed and approved by the Board of Research Ethics at the Central Finland Health Care District (Dnro 8U/2016). The patients/participants provided their written informed consent to participate in this study.

## Author Contributions

All authors have made substantial contributions to planning, designing, collecting the data, analysis and interpretation of data, participated in drafting the manuscript and writing and commenting the content, and also approved the version to be submitted in the journal.

## Conflict of Interest

The authors declare that the research was conducted in the absence of any commercial or financial relationships that could be construed as a potential conflict of interest.

## Footnotes

1 WHO, 2017: https://apps.who.int/iris/bitstream/handle/10665/254610/WHO-MSD-MER-2017.2-eng.pdf;jsessionid=31927 89A665A9D4F33D0EC6FCE80C9F8?sequence=1.

2 Findikaattori, 2019: https://findikaattori.fi/fi/76.

3 EADMT Ethical Code, 2010: http://www.eadmt.com/?action=articleandid=22.

4 Finnish Current Care Guidelines, 2016: https://www.kaypahoito.fi/hoi50023.

5 The European Association Dance Movement Therapy, 2013: http://www.eadmt.com/?action=articleandid=22.

6 KELA, 2020: https://www.kela.fi/web/en/rehabilitative-psychotherapy.

7 WHO, 2020. Gender and women’s mental health: https://www.who.int/mental_health/prevention/genderwomen/en/.
